# Novel biomarkers of a peripheral blood interferon signature associated with drug-naïve early arthritis patients distinguish persistent from self-limiting disease course

**DOI:** 10.1038/s41598-020-63757-3

**Published:** 2020-06-01

**Authors:** Attila A. Seyhan, Bernard Gregory, Adam P. Cribbs, Sundeept Bhalara, Yizheng Li, Christine Loreth, Ying Zhang, Yongjing Guo, Lih-Ling Lin, Marc Feldmann, Lynn M. Williams, Fionula M. Brennan, Peter C. Taylor

**Affiliations:** 1 Kennedy Institute of Rheumatology, NDORMS, University of Oxford, Oxford, OX3 7FY United Kingdom; 2 Watford General Hospital, West Herts NHS Trust, Watford, Herts UK; 3 Pfizer, Worldwide Research, Development and Medical, Cambridge, MA USA; 4 Botnar Research Centre, Windmill Road, Headington, Oxford OX3 7LD United Kingdom; 5 Present Address: Joint Program in Cancer Biology, Brown University and Lifespan Cancer Institute and Department of Pathology and Laboratory Medicine, Division of Biology and Medicine, Brown University, Providence, RI 02903 USA; 6 Present Address: Immunology and Inflammation, Sanofi, Paris France

**Keywords:** Immunogenetics, Acute inflammatory arthritis

## Abstract

We profiled gene expression signatures to distinguish rheumatoid arthritis (RA) from non-inflammatory arthralgia (NIA), self-limiting arthritis (SLA), and undifferentiated arthritis (UA) as compared to healthy controls as novel potential biomarkers for therapeutic responsiveness. Global gene expression profiles of PBMCs from 43 drug-naïve patients presenting with joint symptoms were evaluated and differentially expressed genes identified by comparative analysis with 24 healthy volunteers. Patients were assessed at presentation with follow up at 6 and 12 months. Gene ontology and network pathway analysis were performed using DAVID Bioinformatics Resources v6.7. Gene expression profiles were also determined after disease-modifying anti-rheumatic drug (DMARD) treatment in the inflammatory arthritis groups (i.e. RA and UA) and confirmed by qRT-PCR. Receiver operating characteristic (ROC) curves analysis and Area Under the Curve (AUC) estimation were performed to assess the diagnostic value of candidate gene expression signatures. A type I interferon (IFN) gene signature distinguished DMARD-naïve patients who will subsequently develop persistent inflammatory arthritis (i.e. RA and UA) from those with NIA. In patients with RA, the IFN signature is characterised by up-regulation of *SIGLEC1* (*p* = 0.00597) and *MS4A4A* (*p* = 0.00000904). We also identified, *EPHB2* (*p* = 0.000542) and *PDZK1IP1* (*p* = 0.0206) with RA-specific gene expression profiles and elevated expression of the *ST6GALNAC1* (*p* = 0.0023) gene in UA. ROC and AUC risk score analysis suggested that MSA4A (AUC: 0.894, 0.644, 0.720), PDZK1IP1 (AUC: 0.785, 0.806, 0.977), and EPHB2 (AUC: 0.794, 0.723, 0.620) at 0, 6, and 12 months follow-up can accurately discriminate patients with RA from healthy controls and may have practical value for RA diagnosis. In patients with early inflammatory arthritis, *ST6GALNAC1* is a potential biomarker for UA as compared with healthy controls whereas *EPHB2*, *MS4A4A, and particularly PDZK1IP1* may discriminate RA patients. *SIGLEC1* may also be a useful marker of disease activity in UA.

## Introduction

A genetic basis for rheumatoid arthritis (RA) has long been established most notably in the association of HLA-Dw4 with RA^1,2^. Some reports have estimated the genetic contribution to account for up to two-thirds of RA susceptibility^3^ with the HLA locus contributing 30 to 50%^4^. Recent efforts using high-density genetic mapping have identified new genetic susceptibility loci for RA^5,6^. A recent genome-wide association study meta-analysis identified 42 significant novel RA risk loci, bringing the total to 101^6^. Further in silico analysis based on functional annotation, cis-acting expression quantitative trait loci, pathway analyses and genetic overlap with human primary immunodeficiency, haematological cancer somatic mutations and knockout mouse phenotypes identified 98 biological candidate genes at these 101 risk loci. Among these new susceptibility loci, a number of which were associated with RA overall and several of which were specifically associated with disease that was anti-citrullinated peptide antibody positive^5^. Furthermore, many of these genes are the targets of approved therapies for RA suggesting that drugs approved for other indications may be repurposed for the treatment of RA. Although candidate gene-based and comprehensive in silico genome-wide association studies (GWAS) study meta-analysis have contributed to defining this heritability, identifying more than 98 biological candidate genes at 101 non-HLA RA susceptibility loci^6,7^, very little is known about how these genetic risks influence disease development or treatment response in human studies.

The 1987 American College of Rheumatology (ACR) classification criteria were developed to permit recruitment of relatively homogeneous patient phenotypes into trials but they were not designed to identify patients with early stage disease. Evidence now supports subdivision of the RA syndrome into two major subsets based on the presence or absence of autoantibodies to citrullinated protein antigen (ACPA)^8^. The introduction of new ACR/European League Against Rheumatism (EULAR) classification criteria permit earlier identification and treatment of persistent inflammatory arthritis [i.e. RA and undifferentiated arthritis (UA)]^9,10^ as there is compelling evidence for best outcomes if effective therapy is implemented early^11^. With the increasing range and availability of effective targeted therapies in the clinic and in development^12^, a contemporary challenge is to predict which patients will benefit from early intervention with drugs of particular mechanism of action.

Gene expression profiling studies in PBMC^13^ and validated in synovial tissue^14^ offer a non-biased, complementary approach to GWAS studies and may confirm risk associations at the level of gene expression associated with pathology, as well as identifying biomarkers of therapeutic response to targeted therapies^15^. However, there are only a few studies reported in drug-naïve early arthritis patient cohorts. In one study, Pratt *et al*.^16^ identified IL-6-mediated STAT-3 signalling in purified peripheral blood CD4 T cells of 173 patients who manifested the earliest clinical phase of RA, which is most noticeable in seronegative disease. However, the role of this pathway in disease pathogenesis awaits further clarification. In another study, Cooles *et al*.^17^ conducted phenotypic and transcriptomic profiling of peripheral blood plasmacytoid and conventional dendritic cells in early drug-naïve RA and compared findings with healthy controls; however transcriptional analysis involved a targeted immunology-related gene panel not a genome scale analysis.

Here we report the results of gene expression signatures of a longitudinal study in well-defined clinical cohorts of drug-naïve, early inflammatory arthritis patients to distinguish RA from non-inflammatory arthralgia (NIA), self-limiting arthritis (SLA, and UA as compared to healthy controls as novel potential biomarkers for therapeutic responsiveness.

Comparisons of gene expression levels from baseline to post-treatment at 6 months and at 12 months were performed. Uniquely, our study included two comparator groups which allowed us to discriminate gene expression profiles unique to drug-naïve RA and/or UA patients as distinct from those with SLA and NIA.

## Methods

### Subjects and study design

This study was performed in compliance with the Declaration of Helsinki. Consecutive, DMARD- and corticosteroid-naïve patients with peripheral joint symptoms presenting to the Charing Cross Hospital Early Arthritis Clinic were recruited with approval of the Riverside Research Ethics Committee (RREC Ref #07/H0706/127). A total of 43 subjects were enrolled and a sample of venous blood was drawn at their first clinic visit and all provided written, informed consent. Patients were assessed by a consultant rheumatologist at presentation with follow up at 6 and 12 months when additional blood samples were taken. Study subjects were assigned to prospective or retrospective classification categories based on clinical findings at presentation and follow up. Sample collection commenced before publication of the 2010 ACR/EULAR classification criteria. The classification of RA was therefore assigned prospectively to those patients fulfilling 1987 American College of Rheumatology criteria^18^. Where there was definite evidence of peripheral inflammatory arthritis that persisted during the first 6 months follow-up, and patients fulfilled neither criteria for RA nor other inflammatory joint disease (e.g. ankylosing spondylitis, sarcoidosis), the patient was assigned a retrospective classification of UA. Where there was definite peripheral arthritis at presentation but the patient was deemed to be in remission by 6 months follow-up without the need for DMARDs, the patient was assigned a retrospective classification of SLA. 23 patients in this analysis had persistent inflammatory arthritis [i.e. 14 RA and 9 UA. Another 7 subjects had SLA]. In addition, a fourth group of 13 patients presenting with peripheral joint pain but with no clinical, serological or imaging evidence of inflammation over 1 year of follow-up were assigned a retrospective classification of NIA.

A single blood sample was also collected from 24 healthy laboratory staff volunteers with no previous diagnosis of chronic inflammatory or autoimmune diseases and served as a reference for the clinical cohorts. Clinical characteristics of all study participants are summarised in Table 1. Disease activity scores (DAS28), serology and DMARD use for the RA patients analysed in the longitudinal arm of this study are shown in Table 2.

**Table 1:** Characteristics of the patient groups and healthy controls.

Category	Healthy Controls n = 25	Persistent Arthritis RA UA n = 14 n = 9		Self-limiting Arthritis (SLA) n = 7	Non-Inflammatory Arthralgia (NIA) n = 13
Mean Age, years	45	62	45	40	51
Sex, female:male	17:8	11:3	8:1	4:3	12:1
ACPA^+^ n (%)	N.D	8 (57)	1 (11)	0 (0)	0 (0)
Rheumatoid factor^+^ n (%)	N.D	10 (71)	1 (11)	1 (14)	2(15)

**Table 2:** Characteristics of inflammatory arthritis patients with demographics, disease activity scores at first presentation and subsequent DMARD use.

Patient ID	Sex	Age, years	RhF+/−	ACPA+/−	ESR mm/hr	CRP mg/L	ANA+/−	DAS28 0–6–12 months	DMARD
RA1	F	34	**−**	**−**	8	ND	+	ND - 4.29–1.7	MTX
RA2	F	58	**+**	**+**	49	ND	-	ND	MTX
RA3	F	77	**+**	**-**	48	ND	ND	7.04-3.1 - ND	MTX, Depo, Naproxen
RA4	F	33	**−**	**−**	27	5	**−**	ND	MTX, Pred
RA5	F	58	**+**	**+**	42	75	+	ND − 5.02 - ND	MTX, HCC, Pred
RA6	M	71	**−**	**−**	39	30	**−**	6.39–2.3–3.1	MTX, HCC, Pred
RA7	F	60	**+**	**+**	67	33	+	4.44–5.8–2.2	MTX
RA8	F	67	**+**	**+**	26	5	ND	5.59–3.75 - ND	MTX
RA9	F	86	**−**	**−**	49	52	**−**	5.53–2.9 - ND	MTX, Pred, SSZ
RA10	M	64	**+**	**+**	8	6	**−**	3.06–2.9 - ND	SSZ, HCQ
RA11	F	69	**+**	**+**	43	19	+	5.56 - ND - ND	MTX, HCQ
RA12*	F	28	**+**	**−**	60	30	+	6.88 -	
RA13	F	68	**+**	**+**	ND	6	**−**	ND	MTX
RA14*	M	43	**+**	**+**	5	5	ND	4.72 - ND - ND	Steroids
UD-A1	F	26	**−**	**−**	11	2		4.73 - ND - ND	Depo, HCC
UD-A2	F	51	**−**	**+**	77	ND	+	ND	HCC
UD-A3	F	45	**−**	**−**	8	2	**−**	ND	Depo, Diclofenac, HCCL
UD-A4	F	52	**+**	**−**	17	2	**−**	ND	Depo, SSZ
UD-A5	M	56	ND	**−**	21	26	**−**	4.63–2.1 - ND	MTX, Steroids
UD-A6	F	41	**-**	ND	ND	5	+	ND	Depo, HCC
UD-A7	F	45	ND	**−**	10	2	ND	ND	Not treated
UD-A8	F	20	**−**	ND	24	7	**−**	4.18 - ND -ND	Not treated
UD-A9	F	63	**−**	**−**	33	7	**−**	4.87 - ND - ND	Steroids

Abbreviations. Rhf, rheumatoid factor; ACPA, anti-citrullinated peptide antibody; CRP, C-reactive protein; ANA, anti-nuclear antibody; Depo, Depomedrone; Pred, Prednisolone; HCC, hydrocortisone; SSZ, sulphasalazine; HCQ, hydroxychloroquinine; MTX, methotrexate; ND, not documented. *patients who left the study after first presentation.

### Blood Sampling and RNA isolation for microarray hybridization and RT-PCR

Samples of venous blood were collected in a CPT Vacutainer cell purification tube (Becton Dickinson, Franklin Lakes, NJ, USA) to isolate PBMCs for gene expression profiling. For RNA extraction PBMCs were separated from whole blood within two hours of blood draw, according to the manufacturer’s instructions and described previously^13^ then snap frozen and stored in liquid nitrogen until processed for analysis. Total RNA isolation was performed using QIA RNeasy mini kit (Qiagen, Valencia, CA) according to the manufacturer’s recommendations. Samples were subjected to on-column DNase treatment to remove potential contaminating DNA. Eluted RNA was quantified using a ND-8000 Spectrophotometer (Nanodrop, Wilmington, DE). RNA quality was accessed on Agilent Bioanalyzer (Agilent, Santa Clara, CA). 100 ng total RNA were used to generate biotin labelled cRNA using 3’ IVT express kit (Affymetrix, Santa Clara, CA) according to manufacturer’s instructions. The kit uses an oligo T7 primer in a reverse transcription reaction followed by *in vitro* transcription reaction with biotin labelled UTP and CTP.

### Microarray hybridization

10 μg of cRNA were fragmented and hybridized to GeneChip(R) Human Genome U133 plus 2 array (Affymetrix, Santa Clara, CA) representing over 47, 000 RNA transcripts and variants (quality control and pre-processing of the microarray were conducted according to the manufacturers recommendation). Hybridized arrays were stained according to the manufacturers’ protocols (Affymetrix, Santa Clara, CA) on a Fluidics Station 450 and scanned on an Affymetrix scanner 3000 7 G. All array images were inspected for defects and quality via Expressionist Refiner Array (Genedata, Switzerland).

### Data filtering and analysis

Cel files containing raw gene expression intensity data were imported into ArrayStar microarray analysis software (DNASTAR, Inc. Madison, WI, USA). In order to minimise the effect of technical variability, the RMT (random matrix theory) method^19^ was carried out to normalise across all chips using chip-included internal controls. Positively transcribed genes were selected if their mean expression levels were at least two fold increased or decreased compared to healthy controls (*p* < 0.05) based on the student’s test. The biological functions of candidate genes were classified using the Database for Annotation, Visualization and Integrated Discovery (DAVID) Bioinformatics Resources v6.7 (The Database for Annotation, Visualization and Intergraded Discovery) and gene ontology analysis were called significant with a false discovery rate (FDR) < 0.05^20^. The microarray data will be submitted to the Gene Expression Omnibus (GEO) public repository.

### Statistical analysis

Differences in mean expression between groups were tested by ANOVA using ArrayStar microarray analysis software (DNASTAR, Inc. Madison, WI, USA). *P*-values < 0.05 were considered significant.

### Quantitative real-time PCR analysis

To measure the expression level of genes of interest, we designed a 48-gene custom Taqman Low Density Array (TLDA, ThermoFisher Scientific, Waltham, MA), including multiple housekeeping genes. To perform RT-qPCR reaction, 100 ng of each RNA sample was first reverse transcribed to cDNA using High Capacity cDNA archive kit (ThermoFisher Scientific, Waltham, MA). Then each cDNA was mixed with 2x TaqMan® Fast Universal PCR Master Mix (ThermoFisher Scientific, Waltham, MA) and 100μl cDNA-PCR mix was loaded onto a TLDA card. TLDA cards were processed on ViiA7 instrument (ThermoFisher Scientific, Waltham, MA) following the manufacturer’s protocol. Normalization of RNA expression data (raw Ct) was based on the delta Ct method. The geometric mean of four housekeeping controls (PGK1, ZNF592, GUSB, and 18 S) was used as the normalizing reference. For statistical analysis the Welch test was applied to the normalized data (delta Ct) in all four clinical groups at baseline compared to healthy control. Differential expression was defined as a minimum 1.5 fold change and a Q value of <0.10 (multiple test correction). The same criteria were applied to filter significant changes in longitudinal comparisons of gene expression levels from baseline to post-treatment at 6 months and for the 6 to 12 months post-treatment interval.

### Receiver Operating Characteristics

Receiver operating characteristic (ROC) curves analysis and Area Under the Curve (AUC) estimation were performed as described in the literature^21–25^. ROC analysis and AUC estimation were used to determine if baseline levels of any of the SIGLEC1, MSA4A, PDZK1IP1, EPHB2, and ST6GALNAC1 gene expression signature scores may discriminate between RA patients at Day 0, 6 months and 12 months follow-up as compared with healthy control.

We also determined if baseline levels of any of these gene expression signature scores may discriminate SLA, NIA, UA, and RA patients from healthy controls.

The sensitivity, specificity, and 95% confidence interval (CI) values were determined at the optimal cut-off value (threshold) from the ROC curve as described^21–25^. Analysis was performed using prism v8.

## Results

### Gene microarray analysis in patients presenting to the early arthritis clinic

**Figure 1:**
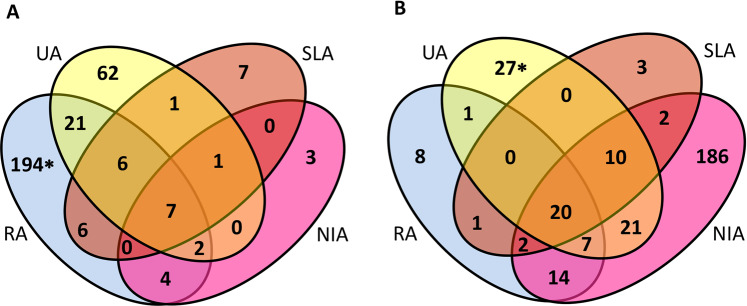
Transcripts expression profiles in all arthritis cohorts. Transcripts displaying a minimum two-fold up-regulation (**A**) or down-regulation (**B**) with respect to healthy controls. *One transcript was up-regulated in RA and down-regulated in UA.

In the NIA cohort as a whole a total of 617 transcripts, representing 508 genes, displayed a 2-fold change compared to the healthy control group, with comparable numbers of more highly-expressed (51%) and under-expressed (49%) transcripts (*p* < 0.05). The distribution of differentially expressed (DE) transcripts was similar within the UA (100 up, 87 down) and SLA (28 up, 38 down) cohorts, whereas in RA most (241 were up-regulated and just 53 down-regulated. Conversely, in the NIA cohort most DE transcripts (262) were down-regulated and 17 up-regulated (Fig. 1).

### Differentially expressed genes unique to persistent arthritis patient groups

To determine DE gene expression profiles unique to persistent arthritis we removed transcripts from the RA and UA groups which were also up co-regulated in the SLA and NIA groups. This identified 277 transcripts (231 annotated genes, 14 uncharacterized) which are potentially uniquely up-regulated in persistent arthritis. Of the 277 DE up-regulated transcripts in the persistent arthritis group, 194 transcripts representing 164 genes (156 annotated, 8 uncharacterized), were only elevated in RA (Additional File 2A), and 62 transcripts representing 61 genes (55 annotated, 6 uncharacterized), were unique to UA (Additional File 2B). One gene, protein S (alpha) (*PROS1*), displayed elevated expression in RA but a down-regulated expression pattern in UA (Additional File 2A). A further group of 21 transcripts, representing 17 genes, were elevated in both groups (Additional File 2C).

Of the total 111 transcripts down-regulated in the persistent arthritis group, only 8 transcripts representing 8 genes, were uniquely down in RA (Additional File 2A) compared to 27 genes only down-regulated in the UA (Additional File 2E) group.

### Ontology enrichment analysis of genes differentially expressed in persistent inflammatory arthritis groups

DE genes unique to the RA and UA cohorts were annotated and categorised according to biological function using gene clustering with DAVID bioinformatics software against a background of all human genes. Gene set enrichment analysis of up-regulated RA-specific genes revealed 151 genes associated with GO terms in 6 clusters of biological processes with an FDR < 0.05. These included secretion via cytoplasmic membrane-bounded vesicles; haemostasis, blood coagulation and the regulation of body fluid levels; response to infection; chemotaxis; active membrane transport and regulation of protein kinase activity (Table 3). A single cluster comprising 13 genes involved in an active immune response was associated with genes up-regulated specifically in the UA group (data not shown). No significant clusters were associated with down-regulated genes in either of the persistent arthritis groups.

**Table 3:** Ontology enrichment analysis of genes differentially up-regulated in RA.

Annotation Cluster 1	Enrichment Score: 7.07	Genes Count (%)	Significance *P* Value	Fold Enrichment	FDR
Category	Term				
GOTERM_CC_ALL	secretory granule	16 (10.88)	3.35 × 10^−11^	10.40	4.21 × 10^−8^
GOTERM_CC_ALL	Vesicle	23 (15.65)	4.72 × 10^−8^	4.02	5.93 × 10^−5^
GOTERM_CC_ALL	cytoplasmic vesicle	22 (14.97)	1.06 × 10^−7^	4.01	1.33 × 10^−4^
GOTERM_CC_ALL	cytoplasmic membrane-bounded vesicle	18 (12.25)	4.15 × 10^−6^	3.83	5.22 × 10^−3^
GOTERM_CC_ALL	membrane-bounded vesicle	18 (12.25)	6.36 × 10^−6^	3.71	7.99 × 10^−3^
**Annotation Cluster 2**	**Enrichment Score: 6.74**				
GOTERM_BP_ALL	Hemostasis	10 (7.48)	3.55 × 10^−8^	11.50	5.95 × 10^−5^
GOTERM_BP_ALL	Coagulation	10 (6.80)	2.68 × 10^−7^	11.07	4.48 × 10^−4^
GOTERM_BP_ALL	blood coagulation	11 (6.80)	2.68 × 10^−7^	11.07	4.48 × 10^−4^
GOTERM_BP_ALL	regulation of body fluid levels	10 (7.48)	4.44 × 10^−7^	8.81	7.43 × 10^−4^
**Annotation Cluster 3**	**Enrichment Score: 5.50**				
GOTERM_BP_ALL	response to other organism	15 (10.20)	2.51 × 10^−7^	5.84	4.19 × 10^−4^
GOTERM_BP_ALL	response to biotic stimulus	16 (10.88)	1.35 × 10^−6^	4.71	2.26 × 10^−3^
**Annotation Cluster 4**	**Enrichment Score: 4.37**				
GOTERM_BP_ALL	Taxis	10 (6.80)	1.15 × 10^−5^	7.06	1.93 × 10^−2^
GOTERM_BP_ALL	Chemotaxis	10 (6.80)	1.15 × 10^−5^	7.06	1.93 × 10^−2^
GOTERM_BP_ALL	locomotory behaviour	12 (8.16)	2.93 × 10^−5^	4.95	4.90 × 10^−2^
**Annotation Cluster 5**	**Enrichment Score: 3.79**				
GOTERM_MF_ALL	P-P-bond-hydrolysis-driven transmembrane transporter activity	9 (6.12)	1.07 × 10^−5^	8.46	1.48 × 10^−2^
GOTERM_MF_ALL	primary active transmembrane transporter activity	9 (6.12)	1.07 × 10^−5^	8.46	1.48 × 10^−2^
**Annotation Cluster 6**	**Enrichment Score: 3.63**				
GOTERM_BP_ALL	positive regulation of protein kinase activity	11 (7.48)	2.69 × 10^−5^	5.57	4.49 × 10^−2^

### RA and UA microarray gene signature responses to treatment regime

**Figure 2:**
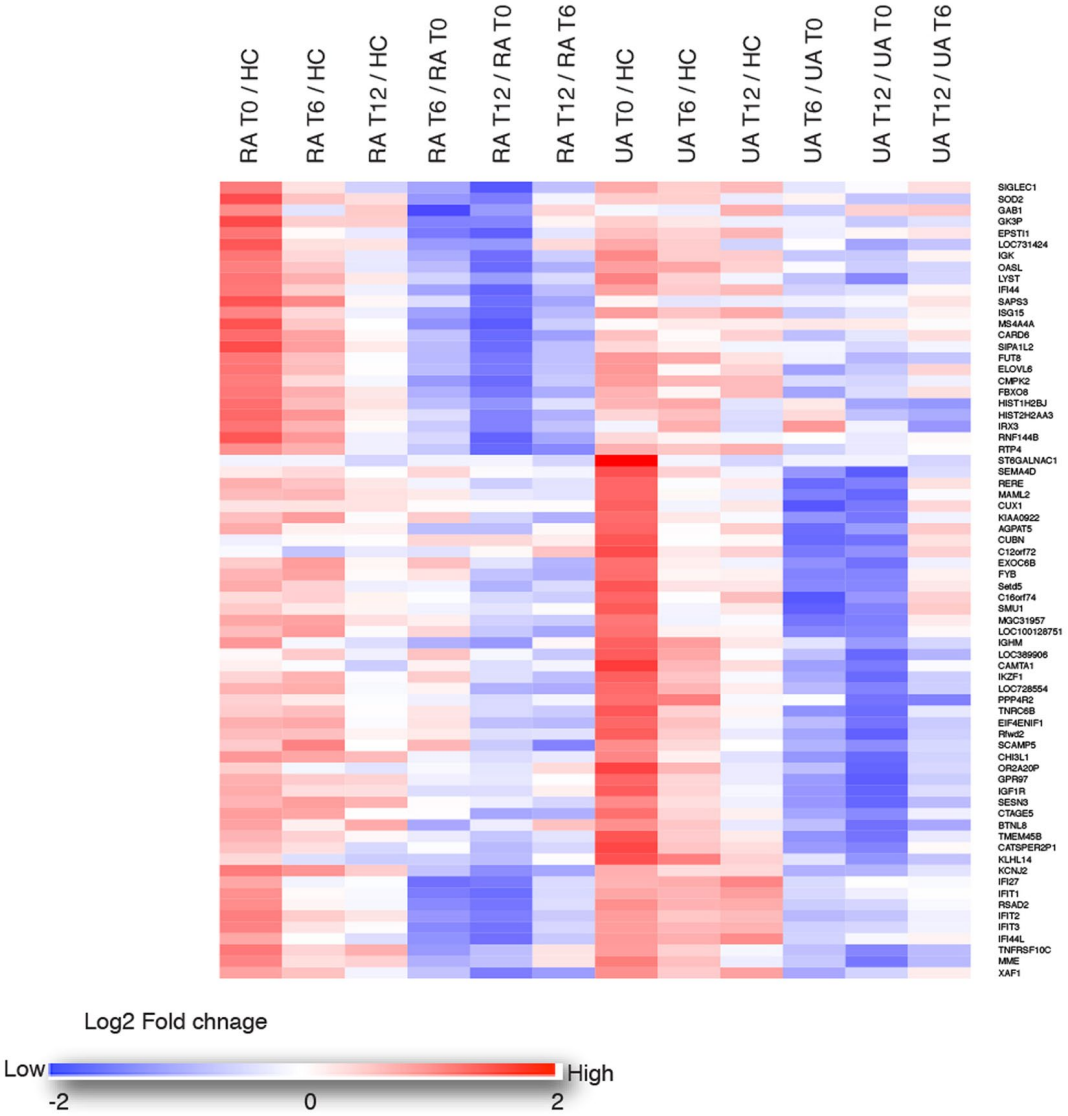
RA and UA gene signature responses to treatment regimes. A heat map representing colour-coded expression levels of differentially expressed (normalised, log2 fold changes) responsive genes in rheumatoid arthritis (RA) and undifferentiated arthritis (UA) patients following baseline (T0), 6 months (T6) and 12 months (T12) of methotrexate or corticosteroid therapy.

To assess the effect of DMARD treatment in RA and UA cohorts, we performed a ratio analysis based on our microarray data. This was expressed as fold-decrease and we considered greater than 2 fold reductions in transcript levels, at either 6 months and/or 12 months after the initiation of treatment with conventional synthetic DMARDs (csDMARDs) +/− steroids, or with steroids alone as detailed in Table 2, to be indicative of a treatment response. By this calculation, a small number of transcripts were found to be lower in RA and UA than in healthy controls or in response to csDMARD therapy. This could be due to the small sample size and/or a small number of transcripts were found to be lower in RA and UA than in healthy controls. Among the few transcripts that were found to be downregulated in RA and UA in response to csDMARD therapy, 25/164 unique RA genes (15%) exhibited at minimum two-fold down regulation post treatment (methotrexate) including 7 of the 8 RA-specific type I interferon response (IFN) genes namely, sialic acid binding Ig-like lectin 1 (*SIGLEC1*), epithelial stromal interaction 1 (breast) (*EPSTI1*), 2′-5′-oligoadenylate synthetase-like (*OASL*), interferon-induced protein 44 (*IFI44*), ISG15 ubiquitin-like modifier (*ISG15*) and membrane-spanning 4-domains, subfamily A, member 4 (*MS4A4A*) and receptor (chemosensory) transporter protein 4 (*RTP4*)(Fig. 2 and Additional File 3A). In the UA cohort, 38/64 (60%) of the uniquely elevated genes demonstrated a treatment response (corticosteroids) (Fig. 2 and Additional File 3B). Among the 17 genes commonly elevated in UA and RA, 10 were attenuated by DMARDs. This category included potassium inwardly-rectifying channel, subfamily J, member 2 (*KCNJ2*) and all 7 of the IFN genes that were only reduced following the RA treatment regimen. Two genes, tumour necrosis factor receptor superfamily, member 10c, decoy without an intracellular domain (*TNFRSF10C*) and membrane metallo-endopeptidase (*MME*) responded specifically to treatment in the UA group (Fig. 2 and Additional File 3C**)**.

### Quantitative validation of selected gene expression profiles

**Figure 3:**
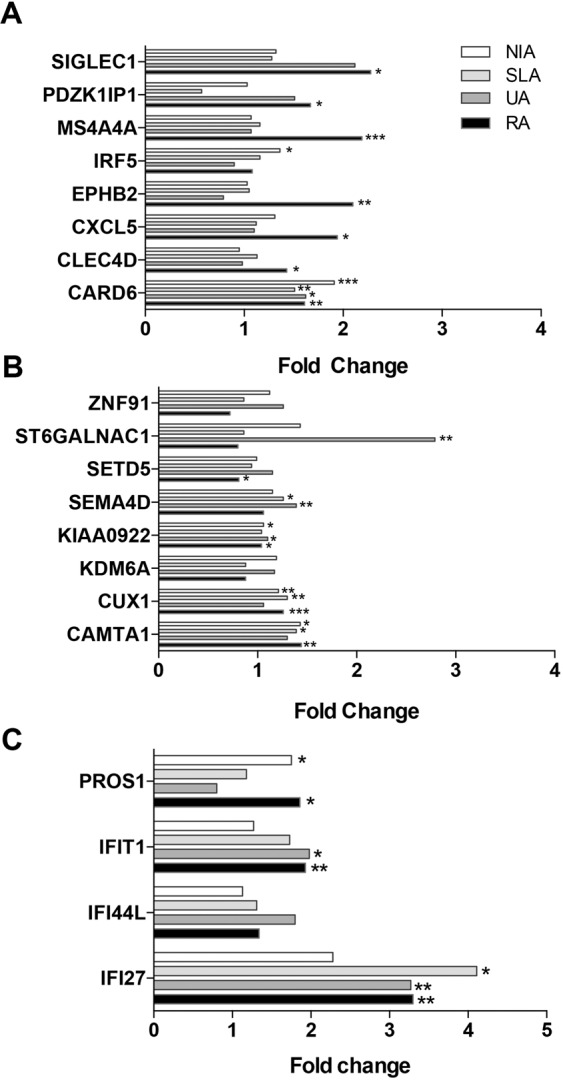
Quantitative baseline expression of 20 selected genes in arthritis cohorts with respect to expression in healthy control. Relative expression of selected gene expression in non-inflammatory arthralgia (NIA), self-limiting arthritis (SLA), undifferentiated arthritis (UA) and rheumatoid arthritis (RA) clinical groups at baseline compared to healthy control, as determined by quantitative RT-PCR. Gene expression profiles identified by microarray analysis as uniquely elevated in RA (**A**) UA (**B**) or co-regulated in both UA and RA (**C**) are shown. Genes with Welch test *p-value* < 0.05 and a Q value of <0.10 were considered statistically significant and are indicated, n = 6 independent donors and bars represent standard error of mean. *P < 0.05, **P < 0.005, ***P < 0.0005.

The expression profiles of 20 genes of biological interest identified by microarray analysis were confirmed by quantitative polymerase chain reaction analysis (qPCR) (Table 4). This panel of genes was chosen on the basis of evidence from the microarray data for disease group-discriminating expression profiles at baseline and/or DMARD-responsiveness (DR) longitudinally with minimum 2 fold change and *p*- value <0.05. Additionally, we also included ST6 (alpha-N-acetyl-neuraminyl-2,3-beta-galactosyl-1,3)-N-acetylgalactosaminide alpha-2,6-sialyltransferase 1) (*ST6GALNAC1*) within our qPCR panel because, while it did not reach significance, it showed a strong > 2 fold regulation. Therefore, profiles indicating gene expression patterns (a) unique to RA (n = 8), including caspase recruitment domain family, member 6 (*CARD6*), C-type lectin domain family 4, member D (*CLECD4*), chemokine (C-X-C motif) ligand 5 (*CXCL5*), EPH receptor B2 (*EPHB2)*, interferon regulatory factor 5 (*IRF5)*, membrane-spanning 4-domains, subfamily A, member 4 (*MS4A4A*), PDZK1 interacting protein 1 (*PDZK1IP1*), sialic acid binding Ig-like lectin 1 (*SIGLEC1)*; (b) unique to UA (n = 8), calmodulin binding transcription activator 1 (*CAMTA1*), cut-like homeobox 1 (*CUX1*), lysine (K)-specific demethylase 6A (*KDM6A*), KIAA0922 (*KIAA0922*), sema domain, immunoglobulin domain (Ig), transmembrane domain (TM) and short cytoplasmic domain, (semaphorin) 4D (*SEMA4D*), SET domain containing 5 (*SETD5*), *ST6GALNAC1*, zinc finger protein 91 (*ZNF91*); and (c) those common all inflammatory arthritis groups including interferon-induced protein with tetratricopeptide repeats 1 (*IFIT1*), interferon, alpha-inducible protein 27 (*IFI27*), interferon-induced protein 44-like (*IFI44L*) were chosen for validation. We also included the protein S alpha (*PROS1*) gene, which exhibited divergent patterns of expression in RA versus UA on the microarray. The data for this gene panel is shown in Table 4 and Fig. 3.

**Table 4:** Quantitative baseline expression of 20 genes differentially expressed in persistent arthritis [i.e. rheumatoid arthritis (RA) and undifferentiated arthritis (UA)] relative to healthy control group.

Gene	RA T0/HC			UA T0/HC			SLA T0/HC			NIA T0/HC		
	T0	*P*-Value	BH Q-Value	T0	*P*-Value	BH Q-Value	T0	*P*-Value	BH Q-Value	T0	*P*-Value	BH Q-Value
CAMTA1	1.44	4.23 × 10^−3^	1.16 × 10^−2^	1.30	3.66 × 10^−1^	6.05 × 10^−1^	1.39	1.02 × 10^−2^	5.43 × 10^−2^	1.43	3.29 × 10^−2^	1.05 × 10^−1^
CARD6	1.61	1.54 × 10^−3^	5.29 × 10^−3^	1.62	1.55 × 10^−2^	9.27 × 10^−2^	1.51	2.75 × 10^−3^	2.64 × 10^−2^	1.91	4.39 × 10^−4^	7.03 × 10^−3^
CLEC4D	1.43	1.60 × 10^−2^	3.34 × 10^−2^	0.98	2.72 × 10^−1^	4.98 × 10^−1^	1.13	6.66 × 10^−1^	7.43 × 10^−1^	0.95	4.52 × 10^−1^	6.03 × 10^−1^
CUX1	1.25	5.26 × 10^−5^	6.31 × 10^−4^	1.06	1.11 × 10^−1^	3.13 × 10^−1^	1.30	1.59 × 10^−3^	2.54 × 10^−2^	1.21	2.04 × 10^−3^	1.63 × 10^−2^
CXCL5	1.94	1.03 × 10^−2^	2.35 × 10^−2^	1.10	9.59 × 10^−1^	9.91 × 10^−1^	1.12	5.39 × 10^−1^	6.82 × 10^−1^	1.31	3.11 × 10^−1^	4.34 × 10^−1^
EPHB2	2.10	5.42 × 10^−4^	2.46 × 10^−3^	0.79	9.91 × 10^−1^	9.91 × 10^−1^	1.05	5.40 × 10^−1^	6.82 × 10^−1^	1.03	9.94 × 10^−1^	9.97 × 10^−1^
IFI27	3.30	3.90 × 10^−3^	1.16 × 10^−2^	3.27	2.48 × 10^−3^	2.98 × 10^−2^	4.11	1.98 × 10^−2^	8.65 × 10^−2^	2.28	5.42 × 10^−2^	1.45 × 10^−1^
IFI44L	1.34	1.04 × 10^−1^	1.46 × 10^−1^	1.80	4.23 × 10^−2^	1.84 × 10^−1^	1.31	3.97 × 10^−1^	5.77 × 10^−1^	1.13	8.87 × 10^−1^	9.97 × 10^−1^
IFIT1	1.93	4.35 × 10^−3^	1.16 × 10^−2^	1.98	1.80 × 10^−2^	9.58 × 10^−2^	1.73	1.20 × 10^−1^	2.88 × 10^−1^	1.27	6.14 × 10^−2^	1.55 × 10^−1^
IRF5	1.08	6.83 × 10^−1^	7.20 × 10^−1^	0.90	8.26 × 10^−1^	9.91 × 10^−1^	1.16	1.75 × 10^−1^	3.32 × 10^−1^	1.36	1.80 × 10^−3^	1.63 × 10^−2^
KDM6A	0.88	1.77 × 10^−1^	2.29 × 10^−1^	1.17	4.79 × 10^−2^	1.91 × 10^−1^	0.88	3.11 × 10^−1^	4.98 × 10^−1^	1.19	6.58 × 10^−2^	1.58 × 10^−1^
KIAA0922	1.04	1.90 × 10^−2^	3.67 × 10^−2^	1.10	1.30 × 10^−2^	9.27 × 10^−2^	1.04	5.49 × 10^−2^	1.88 × 10^−1^	1.06	8.22 × 10^−3^	4.51 × 10^−2^
MS4A4A	2.19	9.04 × 10^−6^	1.45 × 10^−4^	1.07	6.38 × 10^−1^	9.01 × 10^−1^	1.16	2.48 × 10^−1^	4.42 × 10^−1^	1.07	8.83 × 10^−2^	1.70 × 10^−1^
PDZK1IP1	1.67	2.06 × 10^−2^	3.74 × 10^−2^	1.51	8.57 × 10^−1^	9.91 × 10^−1^	0.57	1.18 × 10^−1^	2.88 × 10^−1^	1.03	6.90 × 10^−1^	8.27 × 10^−1^
PROS1	1.86	1.91 × 10^−2^	3.67 × 10^−2^	0.80	3.97 × 10^−1^	6.14 × 10^−1^	1.18	1.12 × 10^−1^	2.88 × 10^−1^	1.75	8.46 × 10^−3^	4.51 × 10^−2^
SEMA4D	1.06	1.01 × 10^−1^	1.46 × 10^−1^	1.39	1.60 × 10^−3^	2.98 × 10^−2^	1.26	1.19 × 10^−2^	5.72 × 10^−2^	1.15	5.34 × 10^−2^	1.45 × 10^−1^
SETD5	0.81	2.10 × 10^−2^	3.74 × 10^−2^	1.15	1.75 × 10^−1^	4.21 × 10^−1^	0.94	7.31 × 10^−1^	7.80 × 10^−1^	0.99	9.19 × 10^−1^	9.97 × 10^−1^
SIGLEC1	2.28	5.97 × 10^−3^	1.51 × 10^−2^	2.12	6.34 × 10^−2^	2.34 × 10^−1^	1.28	8.52 × 10^−2^	2.40 × 10^−1^	1.32	5.58 × 10^−1^	7.23 × 10^−1^
ST6GALNAC1	0.80	6.90 × 10^−1^	7.20 × 10^−1^	2.79	2.30 × 10^−3^	2.98 × 10^−2^	0.86	6.87 × 10^−1^	7.49 × 10^−1^	1.43	1.00 × 10^−1^	1.85 × 10^−1^
ZNF91	0.72	5.82 × 10^−2^	9.42 × 10^−2^	1.26	1.01 × 10^−1^	3.04 × 10^−1^	0.86	3.47 × 10^−1^	5.34 × 10^−1^	1.12	7.83 × 10^−2^	1.63 × 10^−1^

T0, time at baseline.

### Subgroup discriminating genes at first presentation

Quantitative PCR confirmed preferential expression of many genes in RA over UA and SLA. These included *SIGLEC1* (x 2.28 FC, *p* = 0.00597), *EPHB2* (2.1 × FC, *p* = 0.000542), *MS4A4A* (2.19 × FC, *p* = 0.00000904), *CXCL5* (x 1.94 FC, *p* = 0.0103) and *PDZK1IP1* (x 1.67 FC, *p* = 0.0206). However, although *PROS1* distinguished RA (1.86 × FC, *p* = 0.0191) from UA and SLA, significant level of this gene were also detected in the NIA cohort (1.75 × FC, *p* = 0.00846). Only one gene, *ST6GALNAC1* distinguished UA (2.79 FC, *p* = 0.0023) from the RA and SLA groups (Table 4).

### An interferon gene signature is common to all inflammatory arthritis groups

A feature of the microarray gene signature in the persistent arthritis groups was the presence of an IFN gene signature. This was more prominent in the RA cohort where 8 IFN genes, including *EPSITI1*, *IFI44*, *ISG15*, *MS4A4A*, *OASL*, *RTP4*, serpin peptidase inhibitor, clade G (C1 inhibitor), member 1 (*SERPING1*) and *SIGLEC1*, were uniquely overexpressed (Additional File 2A). A further 8 DE genes in this category, including DEAD (Asp-Glu-Ala-Asp) box polypeptide 58 (RIG-I) (*DDX58)*, *IFI27*, *IFI44L*, interferon-induced protein with tetratricopeptide repeats 1, 2 and 3 (*IFIT1*, *IFIT2*, *IFIT3*), radical S-adenosyl methionine domain containing 2 (*RSAD2*) and XIAP associated factor 1 (*XAF1*), were elevated in both RA and UA cohorts (Additional File 2C). We also noted the expression of several prototypical IFN genes which approached the cut-off in SLA but not NIA, including *IFIT1* (x1.88 FC), *IFI27* (x1.64 FC) and *IFI44L* (x 1.76 FC) (Additional File 2C). This suggested that an interferon response might also be a feature of self-limiting arthritis patients. qPCR validation confirmed elevated *IFI27* expression (x 4.11 FC, *p* = 0.0198) in SLA and persistent arthritis groups. However, the profiles of other IFN response genes tested displayed a more disease-specific pattern such that *IFIT1* only reached statistical significance in RA (x 1.93 FC, *p* = 0.00435) and UA (x 1.98 FC, *p* = 0.0180) and significant levels of *SIGLEC1* (x 2.28 FC, *p* = 0.00597) and *MS4A4A* (x 2.19 FC, *p* = 0.00000904) were restricted to the RA cohort (Fig. 3 and Table 4).

### Genes responding to conventional synthetic DMARDs (csDMARD) treatment

Validation of gene profiles longitudinally using the same 2-fold filter criteria for qPCR revealed that only a modest effect of csDMARD treatment. This was indicated by significant trends in the reduction in the expression of IFN genes *MS4A4A* and *IFIT1* at 6 months in the RA treatment regimen that did not pass the filter. Similar reductions in *IFI27* and *SIGLEC1* levels approached the filter cut-off, but these changes were not significant. In the UA treatment group, a non-significant trend to reduced expression was observed at 12 months for *IFI27*, *SIGLEC1*, *IFI44L* and *PDZK1IP1* (Additional File 4).

### Diagnostic value for five genes for RA Identification

**Figure 4:**
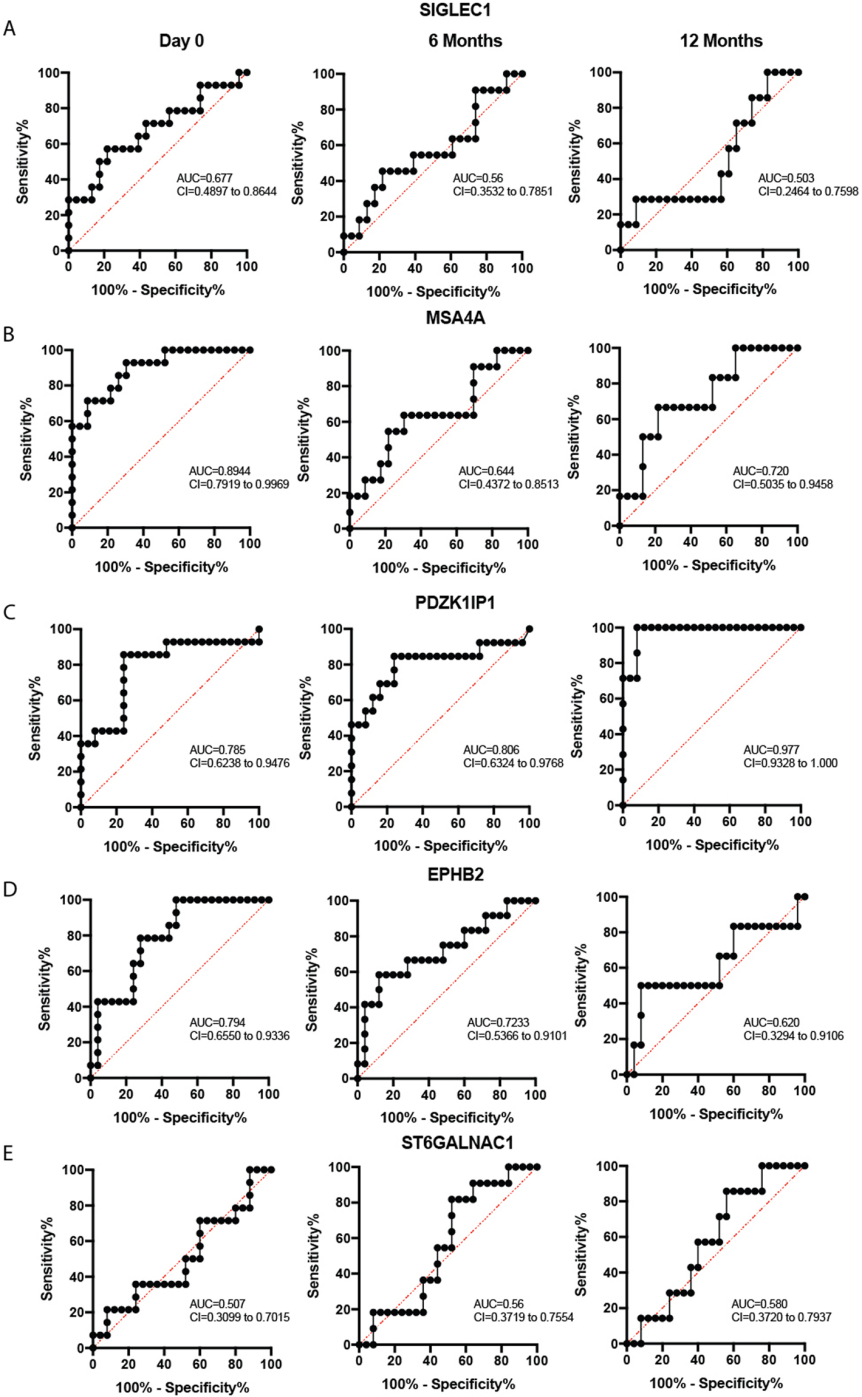
Receiver operating characteristics showing the average predictive performance for Rheumatoid Arthritis. The specificity and sensitivity rate for 5 genes showing the Area Under the Curve (AUC) for patients presenting to the clinic with a diagnosis of RA at Day 0, 6 Months and 12 months follow-up, when compared to healthy individuals. Receiver operating characteristic curves of (**A**) SIGLEC1, (**B**) MSA4A, (**C**) PDZK1IP1, (**D**) EPHB2 and (**E**) ST6GALNAC1 are presented.

**Figure 5:**
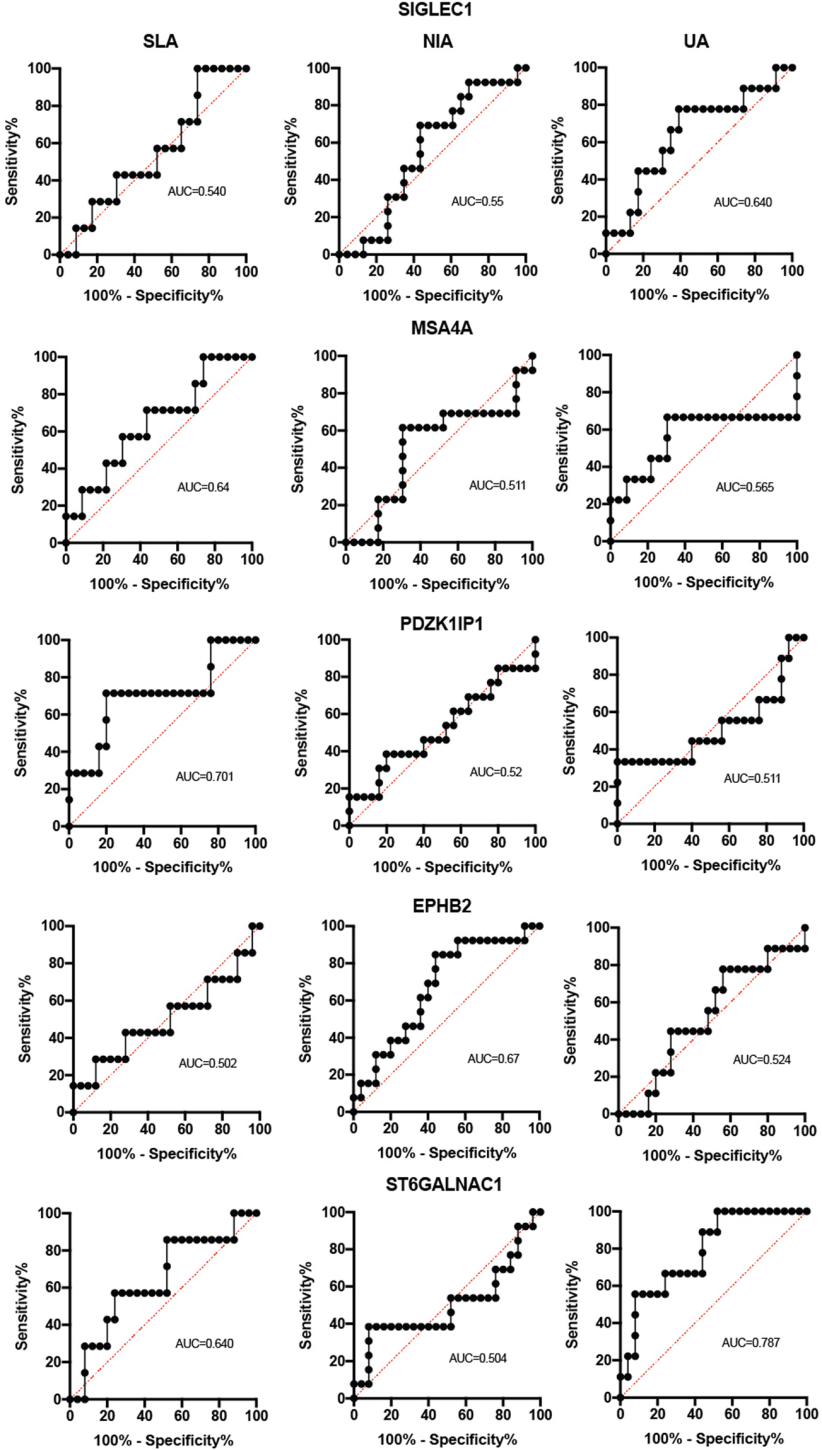
Receiver operating characteristics showing the average predictive performance for self-limiting arthritis (SLA), Non-inflammatory arthritis (NIA) and Undifferentiated arthritis (UA). This figure is similar to Fig. 4 and shows the specificity and sensitivity rate for 5 genes showing the Area Under the Curve (AUC) for patients presenting the clinic with Self Limiting Arthritis, Non-inflammatory arthritis and Undifferentiated arthritis, when compared to healthy individuals. Receiver operating characteristic curves of (**A**) SIGLEC1, (**B**) MSA4A, (**C**) PDZK1IP1, (**D**) EPHB2 and (**E**) ST6GALNAC1 are presented.

To further evaluate the ability of PBMC five gene expression signatures to distinguish RA patients at baseline and 6 and 12 month follow up and healthy controls (Fig. 4) and SLA, NIA, and UA from healthy controls (Fig. 5), we performed ROC curve analysis. As described in the literature^25^, we used the following guide for classifying the accuracy of our genes. An AUC value of 0.5 provides no information for classification, while a value of 1 indicates correct classification. A test with AUC between 0.90 and 1.00 has excellent discrimination ability, AUC from 0.80 to 0.90 has decent discrimination ability, AUC from 0.70 to 0.80 has fair discrimination ability, AUC from 0.60 to 0.70 has poor discrimination ability, and AUC from 0.50 to 0.60 has fail discrimination ability^25^. ROC analysis and AUC estimation showed that baseline levels of any of MSA4A, PDZK1IP1, and EPHB2 gene expression signature scores may discriminate between RA patients at Day 0, 6 months and 12 months follow-up as compared with healthy controls.

As shown in (Fig. 4), ROC analysis performed using signature scores of these five genes showed AUC values as discriminatory ability for SIGLEC1 (Day 0, 0.677, poor score; 6 Months, 0.56, fail score; 12 months, 0.503, fail score); MSA4A (Day 0, 0.8944, good score; 6 Months, 0.644, poor score; 12 months, 0.720, fair score); PDZK1IP1 (Day 0, 0.785, fair score; 6 Months, 0.806, good score; 12 months, 0.977, excellent score); EPHB2 (Day 0, 0.794, fair score; 6 Months, 0.723, fair score; 12 months, 0.620, poor score); and finally ST6GALNAC1 (Day 0.507, fail score; 6 Months, 0.560, fail score; 12 months, 0.580, fail score).

On the other hand, ROC analysis performed using signature scores of these five genes showed no significant discriminatory ability for SIGLEC1, MSA4A, and PDZK1IP1, EPHB2 between SLA, NIA, and UA as compared with healthy control (Fig. 5). Only PDZK1IP1 showed a fair score (0.701) between SLA and healthy control and EPHB2 showed poor score (0.670) between NIA and healthy control and ST6GALNAC1 signature scores were fair (0.787) for discriminating UA from healthy control and poor (0.640) for discriminating SLA from healthy control (Fig. 5).

## Discussion

Our study compared gene expression profiles in patient cohorts who were drug-naïve at presentation, subsequently developing either persistent arthritis or SLA, with reference to controls with arthralgia [i.e. NIA] but no evidence of inflammatory joint disease, and with healthy individuals. Two subgroups were defined with persistent disease; those with inflammatory arthritis remaining undifferentiated [i.e. UA] over the observation period that received steroid treatment and those fulfilling 1987 classification for RA where 10 of 12 patients were prescribed methotrexate. Microarray analysis at baseline revealed distinct and overlapping gene expression patterns in both arthritis subgroups that were unique to the persistent inflammatory arthritis group as a whole. The UA group exhibited signs of an active adaptive immune response and was characterised by a cluster of immunoglobulin genes with raised expression. In the RA group, cellular and biological processes including protein kinase activity, secretion, chemotaxis, response to infection, haemostasis and blood coagulation were elevated.

Using quantitative PCR analysis, we validated disease subset-specific expression patterns of several candidate genes identified by microarray analysis. Only one gene, *ST6GALNAC1*, with a minimum 1.5-fold change and a Q value of <0.1, met our criteria in the undifferentiated arthritis cohort. *ST6GALNAC1* codes for a sialic acid transferase, and has not previously been associated with UA. Sialyltransferases add sialic acid to the terminal portions of glycolipids or to the N- or O-linked sugar chains of glycoproteins. Glycosylation of proteins affects cell-cell interaction, interactions with the matrix, and the functions of many plasma proteins. Micro heterogeneity in glycosylation of IgG, ACPA and many other plasma proteins have been widely studied and are implicated in the pathogenesis of RA^26–28^. However a definitive role of *ST6GALNAC1* has still to be elucidated, although it has been shown to negatively regulate P-selectin function by modification of the glycosylation status of the O-linked glycans at the N-terminus of the leukocyte cell-surface molecule P-selectin glycoprotein ligand-1^29^. Clearly given *ST6GALNAC1* met our strict criterion its role in arthritis clearly warrants further biochemical analysis.

When patients present to an early arthritis clinic, if they do not meet classification criteria for RA, it can be a challenge to confidently identify patients who will eventually follow the disease course of RA, and therefore benefit from prompt intervention with csDMARDs. In this study, ROC and AUC risk score analysis suggested that MSA4A, PDZK1IP1 and EPHB2 at first presentation can discriminate patients with RA from healthy controls and may therefore have practical value for RA diagnosis.

In contrast, ROC analysis performed using signature AUC scores showed no significant discriminatory ability for SIGLEC1, MSA4A, and PDZK1IP1, EPHB2 between SLA, NIA, and UA as compared with healthy control (Fig. 5). However, PDZK1IP1 showed a fair AUC score (0.701) between SLA and healthy control and EPHB2 showed poor AUC score (0.670) between NIA and healthy control. ST6GALNAC1 signature AUC scores were fair (0.787) for discriminating UA from healthy control and poor (0.640) for discriminating SLA from healthy control.

Our data suggest that baseline MSA4A, PDZK1IP1, EPHB2 levels may help to identify RA patients at risk for future progression (Fig. 4). However, since ROC analysis was conducted using the same and relatively limited sample group that was used to construct the gene signatures, an overfitting problem might occur. Therefore, it is warranted to validate our results in sufficiently powered independent cohorts in the future.

We also identified *PDZK1IP1*, *MS4A4A*, and *EPHB2* as genes that have not previously been linked to RA. The plasma membrane protein *PDZK1IP1* (MAP17) gene is also expressed in human carcinoma lines, although the role it plays in resistance to TNF-induced apoptosis^30^ may be of relevance to RA. A more promising RA-discriminating candidate may be the IFNβ-induced gene, *MS4A4A*^31^. This is a member of a large family of structurally similar cell-surface proteins with putative signal transduction functions that includes functionally important B lymphocyte (B cell) marker, CD20^32^. *MS4A4A* was also found to be to be up-regulated in a cohort of DMARD-naïve recent onset juvenile idiopathic arthritis patients^33^ and is absent on normal B lymphocytes^22^. We also identified ephrin B2 receptor with an RA-discriminating profile. A role for erythropoietin-producing human hepatocellular receptors (ephrin receptors) in the aetiology of systemic inflammatory diseases such as RAhas previously been postulated^34^; although evidence to support this speculation has been lacking, high levels of ephrin B1 ligand were reported in peripheral and synovial T lymphocytes (T cells) in a small study with RA patients^35^. Our data demonstrating that *EPHB2* is also elevated in early RA further supports the notion that this system is active once RA becomes established, as opposed to UA.

In agreement with earlier studies we observed an IFN signature in PBMCs in RA^36,37^ where eight genes detected by microarray analysis in our cohort, namely, *IFIT1*, *IFIT2*, *IFI44L*, *RSAD2*, *SERPING1*, *EPSTI1*, *RTP4*, and *ISG15* were identified in an earlier RA gene expression profiling study^38^. However, our study is also the first to report *SIGLEC1* (CD169) expression in the peripheral IFN signature of drug-naïve patients with early RA, which we also confirmed by qPCR. Peripheral expression of *SIGLEC1* is consistent with an earlier report describing co-expression of *SIGLEC1* or Siglec-1 protein in synovial tissue CD68^+^ cells^39^ and a more recent report that demonstrated elevated numbers of Siglec-1^+^ inflammatory monocytes in the periphery of established RA patients^32^.

Activation of an IFN gene signature is a molecular feature shared by many autoimmune diseases including a subset of RA^37^ and is already detectable in the periphery during the preclinical disease phase^38^. Although the clinical relevance of IFN activity remains unclear, it may be that a predominance of TNF over IFN activity or vice versa may promote the development of autoimmune diseases where excess of one cytokine plays a dominant role such as in RA and SLA respectively. Alternatively, IFNs may play a more prominent role in the initiation phase of disease while TNF predominates in the effector phase^40^.

We speculate that differences in the number and magnitude of IFN gene profiles observed between our drug-naïve inflammatory arthritis cohorts may suggest that the spectrum of early inflammatory arthritis from SLA to UA through RA is characterised by progressive escalations in IFN activity whereby *SIGLEC1* expression in particular appears to distinguish persistent arthritis from SLA patients. This hypothesis is supported by studies using *SIGLEC1* knockout animals in experimental models of autoimmune uveoretinitis (EAU) and autoimmune encephalomyelitis (EAE) suggesting a role for *SIGLEC1* in promoting disease development^41^. In this scenario, the capacity of Siglec-1^+^ macrophages to inhibit the proliferation of regulatory T cells (Tregs)^42^ and to induce cell death in Tregs and/or CD4^+^Foxp3^-^ T effector cells (Teffs) has been documented^43^. It is tempting to speculate therefore that increased *SIGLEC1* expression and/or the numbers of circulating Siglec-1^+^ monocyte/macrophages in UA and RA may reflect a homeostatic feedback mechanism engaged to limit increased Treg numbers and/or to regulate the Treg: Teff balance at a critical point in the progression from acute to chronic inflammatory disease status. The notion that Tregs control the transition from acute to chronic inflammation but fail to regulate an established chronic inflammation is supported by evidence in an animal model of self-remitting arthritis^44^. We have also shown in a human *ex vivo* model of RA that Tregs are unable to inhibit proinflammatory cytokine production from more active synovial tissues^45^. However, to understand the role of IFN in inflammatory arthritis and its potential contribution to the pathogenesis of RA, it will be necessary to define the function IFN response genes such as *SIGLEC1* in both disease stage and subtype-specific contexts.

Clinical studies addressing the predictive power of the IFN signature have established the responsiveness of IFN-inducible genes to anti-TNF treatment^38^. While such studies have not defined a consistent response across different therapeutics, the concept of monitoring the IFN response as a predictor of therapeutic response is supported by a recent study which measured IFN activity in RA plasma pre- and post- anti-TNF biologic therapy and reported better EULAR outcomes in patients with a high baseline IFNβ/α ratio^46^. Longitudinal gene microarray profiling in our patient cohorts confirmed DMARD responsiveness of IFN genes, albeit delayed in the undifferentiated arthritis (UA compared to the RA treatment group. Perhaps due the small sample sizes, the reduction in RA-specific IFN genes *SIGLEC1* and *MS4A4A* while significant did not pass the filter stringency in confirmatory qPCR. However, the recent report of Xiong *et al*.^43^, showing a positive correlation for Siglec-1 protein expression on PBMCs with disease activity in established RA, and the parallel decrease in *SIGLEC1* expression with DAS28 after DMARD treatment, suggests that *SIGLEC1* and the more RA-restricted *MS4A4A* identified are both potential biomarkers of disease activity in persistent arthritis. However, *SIGLEC1* has been reported to be a feature of the IFN signature in systemic lupus erythematous^47^, suggesting that its expression alone may not be sufficient to define RA, therefore a combination of *SIGLEC1* and *MS4A4A* expression is more likely to discriminate RA from other inflammatory diseases.

Nevertheless, our study has a number of limitations. First, due to the small sample size for the four subgroups, the large variability of early arthritis, and heterogeneous treatment regimens are the challenges for meaningful analysis and generalizability of our findings to other populations and disease stages. These findings are at best hypotheses generating; therefore, validating these findings in sufficiently powered independent cohorts of each disease subgroup with multiple testing (*i.e*. the Bonferroni adjustment) correction procedure to adjust our statistical confidence measures based on the number of tests performed is warranted to strengthen our finding.

Second, our proposed biomarkers are only at the mRNA level are not validated at the protein level and mechanistic insight into the putative biomarkers is lacking and thus these are obvious limitations of our study. However, for some of these mRNAs, the respective protein products may not be present or detectable in blood; hence quantification of transcript levels may be the better option. Third, because gene expression profiling was carried out in whole PBMCs, there is, therefore, significant chance that differences in the composition of cellular subsets either adds to or diminishes the biomarker potential of these signatures. However, for liquid biopsy to be practical in the clinical setting, expression profiling in whole PBMCs is the best choice, due to its simplicity, high turnaround, and relatively lower cost; not the peripheral blood lymphocyte subsets.

Fourth, the mechanistic insight into the putative biomarkers in relation to the history of disease progression and response to therapies is unknown; therefore, longer-term treatment and follow-up studies to understand mechanisms of action and to better gauge, the clinical utility of these potential biomarkers is warranted.

Nevertheless, results of our pilot and feasibility longitudinal study in well-defined clinical cohorts of drug-naïve, early inflammatory arthritis patients provide some key but yet to be validated potentially useful biomarkers as classifiers to discriminate drug-naïve RA and/or UA patients from those with SLA and NIA as compared to healthy control as well as potential DMARD responsive biomarkers in RA and UA.

## Conclusions

Despite the need for replicating the findings in an independent dataset, this study has potentially identified a type I IFN gene signature in the periphery of drug naïve patients with persistent arthritis and SLA as a promising biomarker. The expression of the immunoregulatory receptor *SIGLEC1* and *MSA4A* is a novel feature which characterizes this IFN signature in persistent arthritis. Finally, we identify *ST6GALNAC1* as a marker of undifferentiated arthritis and *MSA4A, PDZK1IP1* and *EPHB2* whose expression profiles may potentially discriminate untreated early RA from UA and SLA.

## Supplementary information

Dataset 1.

Dataset 2.

Dataset 3.

Dataset 4.

## Data Availability

The datasets supporting the conclusions of this article are included within the article (and its additional files).
