# Supplementary material for: Novel biomarkers of a peripheral blood interferon signature associated with drug-naïve early arthritis patients distinguish persistent from self-limiting disease course
Source: Sci Rep. 2020 Jun 1;10:8830. doi: 10.1038/s41598-020-63757-3 (PMC7264129; doi:10.1038/s41598-020-63757-3)
Supplement: Supplementary file 1 — Dataset 1. [file 41598_2020_63757_MOESM1_ESM.docx]

**Supplementary Table S1**

**Novel biomarkers of a peripheral blood interferon signature associated with drug-naïve early arthritis patients distinguish persistent from self-limiting disease course**

Attila A. Seyhan^3,5,^ **^†^** Bernard Gregory^1,^ **^†^**, Adam P. Cribbs, ^4^, Sundeept Bhalara^2^, Yizheng Li^3^, Christine Loreth^3^, Ying Zhang^3^, Yongjing Guo^3^, Lih-Ling Lin^3^, Marc Feldmann^1^, Lynn M. Williams^1^, Fionula M. Brennan^1#^ and Peter C. Taylor^4^

**Table S1. Characteristics of inflammatory arthritis patients with demographics, disease activity scores at first presentation and subsequent DMARD use**

| **Patient ID** | **Sex** | **Age,**  **years** | **RhF**  **+/-** | **ACPA**  **+/-** | **ESR**  **mm/hr** | **CRP**  **mg/L** | **ANA**  **+/-** | **DAS28 0– 6 – 12 months** | **DMARD** |
| --- | --- | --- | --- | --- | --- | --- | --- | --- | --- |
| RA1 | F | 34 | **-** | **-** | 8 | ND | + | ND - 4.29 - 1.7 | MTX |
| RA2 | F | 58 | **+** | **+** | 49 | ND | - | ND | MTX |
| RA3 | F | 77 | **+** | **-** | 48 | ND | ND | 7.04 - 3.1 - ND | MTX, Depo, Naproxen |
| RA4 | F | 33 | **-** | **-** | 27 | 5 | - | ND | MTX, Pred |
| RA5 | F | 58 | **+** | **+** | 42 | 75 | + | ND - 5.02 - ND | MTX, HCC, Pred |
| RA6 | M | 71 | **-** | **-** | 39 | 30 | - | 6.39 - 2.3 - 3.1 | MTX, HCC, Pred |
| RA7 | F | 60 | **+** | **+** | 67 | 33 | + | 4.44 - 5.8 - 2.2 | MTX |
| RA8 | F | 67 | **+** | **+** | 26 | 5 | ND | 5.59 - 3.75 - ND | MTX |
| RA9 | F | 86 | **-** | **-** | 49 | 52 | - | 5.53 - 2.9 - ND | MTX, Pred, SSZ |
| RA10 | M | 64 | **+** | **+** | 8 | 6 | - | 3.06 - 2.9 - ND | SSZ, HCQ |
| RA11 | F | 69 | **+** | **+** | 43 | 19 | + | 5.56 - ND - ND | MTX, HCQ |
| RA12* | F | 28 | **+** | **-** | 60 | 30 | + | 6.88 - |  |
| RA13 | F | 68 | **+** | **+** | ND | 6 | - | ND | MTX |
| RA14* | M | 43 | **+** | **+** | 5 | 5 | ND | 4.72 - ND - ND | Steroids |
| UD-A1 | F | 26 | **-** | **-** | 11 | 2 | - | 4.73 - ND - ND | Depo, HCC |
| UD-A2 | F | 51 | **-** | **+** | 77 | ND | + | ND | HCC |
| UD-A3 | F | 45 | **-** | **-** | 8 | 2 | - | ND | Depo, Diclofenac, HCCL |
| UD-A4 | F | 52 | **+** | **-** | 17 | 2 | - | ND | Depo, SSZ |
| UD-A5 | M | 56 | ND | **-** | 21 | 26 | - | 4.63 - 2.1 - ND | MTX, Steroids |
| UD-A6 | F | 41 | **-** | ND | ND | 5 | + | ND | Depo, HCC |
| UD-A7 | F | 45 | ND | **-** | 10 | 2 | ND | ND | Not treated |
| UD-A8 | F | 20 | **-** | ND | 24 | 7 | - | 4.18 - ND -ND | Not treated |
| UD-A9 | F | 63 | **-** | **-** | 33 | 7 | - | 4.87 - ND - ND | Steroids |

Abbreviations. RhF, rheumatoid factor; ACPA, anti-citrullinated peptide antibody; CRP, C-reactive protein; ANA, anti-nuclear antibody; Depo, Depomedrone; Pred, rednisolone; HCC, hydrocortisone; SSZ, sulphasalazine; HCQ, hydroxychloroquinine; MTX, methotrexate; ND, not documented. * patients who left the study after first presentation.
